# Genome-wide analysis of DNA methylation patterns in horse

**DOI:** 10.1186/1471-2164-15-598

**Published:** 2014-07-15

**Authors:** Ja-Rang Lee, Chang Pyo Hong, Jae-Woo Moon, Yi-Deun Jung, Dae-Soo Kim, Tae-Hyung Kim, Jeong-An Gim, Jin-Han Bae, Yuri Choi, Jungwoo Eo, Yun-Jeong Kwon, Sanghoon Song, Junsu Ko, Young Mok Yang, Hak-Kyo Lee, Kyung-Do Park, Kung Ahn, Kyoung-Tag Do, Hong-Seok Ha, Kyudong Han, Joo Mi Yi, Hee-Jae Cha, Byung-Wook Cho, Jong Bhak, Heui-Soo Kim

**Affiliations:** Department of Biological Sciences, College of Natural Sciences, Pusan National University, Busan, 609-735 Republic of Korea; TBI, Theragen BiO Institute, TheragenEtex, Suwon, 443-270 Republic of Korea; Genome Resource Center, Korea Research Institute of Bioscience and Biotechnology (KRIBB), 111 Gwahangno, Yuseong-gu, Daejeon 305-806 Republic of Korea; Department of Pathology, School of Medicine, Institute of Biomedical Science and Technology, Konkuk University, Seoul, 143-701 Republic of Korea; Department of Biotechnology, Hankyong National University, Anseong, 456-749 Republic of Korea; Department of Genetics, Human Genetics Institute of New Jersey, Rutgers, the State University of New Jersey, 145 Bevier Rd, Piscataway, NJ 08854 USA; Department of Nanobiomedical Science and WCU Research Center, Dankook University, Cheonan, 330-714 Republic of Korea; Research Center, Dongnam Institute of Radiological & Medical Sciences (DIRAMS), Jwadong-gil 40, Jangan-eup, Gijang-gun, Busan 619-950 Republic of Korea; Department of Parasitology and Genetics, Kosin University College of Medicine, Busan, 602-703 Republic of Korea

**Keywords:** Thoroughbred horse, Jeju horse, Genome-wide DNA methylation, Differential methylated region (DMR), MeDIP-seq

## Abstract

**Background:**

DNA methylation is an epigenetic regulatory mechanism that plays an essential role in mediating biological processes and determining phenotypic plasticity in organisms. Although the horse reference genome and whole transcriptome data are publically available the global DNA methylation data are yet to be known.

**Results:**

We report the first genome-wide DNA methylation characteristics data from skeletal muscle, heart, lung, and cerebrum tissues of thoroughbred (TH) and Jeju (JH) horses, an indigenous Korea breed, respectively by methyl-DNA immunoprecipitation sequencing. The analysis of the DNA methylation patterns indicated that the average methylation density was the lowest in the promoter region, while the density in the coding DNA sequence region was the highest. Among repeat elements, a relatively high density of methylation was observed in long interspersed nuclear elements compared to short interspersed nuclear elements or long terminal repeat elements. We also successfully identified differential methylated regions through a comparative analysis of corresponding tissues from TH and JH, indicating that the gene body regions showed a high methylation density.

**Conclusions:**

We provide report the first DNA methylation landscape and differentially methylated genomic regions (DMRs) of thoroughbred and Jeju horses, providing comprehensive DMRs maps of the DNA methylome. These data are invaluable resource to better understanding of epigenetics in the horse providing information for the further biological function analyses.

**Electronic supplementary material:**

The online version of this article (doi:10.1186/1471-2164-15-598) contains supplementary material, which is available to authorized users.

## Background

DNA methylation is a stably inherited epigenetic modification in eukaryotes. The regulation and characteristics of the DNA methylation still remain enigmatic, although the importance of it has been demonstrated in many biological processes such as gene expression regulation, genomic imprinting, X chromosome inactivation, maintenance of genomic stability by transposon silencing. It has also been implicated in the development of diseases such as cancer [1–7]. DNA methylation is also essential for the proper differentiation and development of mammalian tissues [8, 9]. For instance, the knockout of genes encoding the DNA-methyltransferase (DNMT) enzymes, which are responsible for *de novo* methylation of DNA, results in embryonic lethality in mice [10, 11]. In mammals, methycytosine is observed mostly at CpG dinucleotides, except for the CpGs in CpG islands [12]. DNA methylation is unevenly distributed in genomes: the intergenic regions, and repetitive elements are usually hypermethylated, while the 5′ and 3′ flanking regions of genes are relatively hypomethylated compared with the intragenic regions [13–15]. Recently, whole genome methylation has been extensively examined in mammalian species [16, 17] due to advanced sequencing technologies.

Previous studies have revealed the patterns of global DNA methylation in a single or few tissues across species [18–23], or in multiple tissues or developmental stages in a single organism [8, 18, 24–28]. The DNA methylation pattern is generally conserved, and through comparative analyses of DNA methylation across mammalian species, it has been suggested to play a role in tissue-specific gene regulation [20]. When tissue-specific differentially methylated regions (T-DMRs) in human and mouse tissues including heart, colon, kidney, testis, spleen, and muscle were compared, they could be distinguished clearly according to the corresponding tissues based on their methylation status [27]. It is probable that there are a large number of potentially important functional differences in methylation levels across species. In primates, relative tissue methylation levels generally differ among species [20]. However, there is insufficient evidence indicating that methylation differences exist at subspecies or breeds level.

Thoroughbred horse (TH) is a horse breed that has been manipulated by humans for improved speed, agility, and endurance in England. THs have been selected for racing ability. Thus the genetic traits related to athletic performance against TH have been extensively studied, including genotyping and transcriptome analysis [29–35]. Jeju horse (JH; a natural monument No. 347) is an indigenous Korean horse, is physically a small and rugged pony [36]. They have been raised for meet source, farm labor, riding, and racing in Jeju Island, South Korea. Detailed genetic characterization of JH is thought to be crucial for the conservation of and for effective breeding strategies of this indigenous animal. Thus, many studies have been performed to analyses phylogenetic relationships, and discovering genetic marker [37–39]. However, until now, there have been no studies associate the traits of Jeju horse with epigenetic patterns. With the advent of next-generation sequencing (NGS) and genome-wide association studies, some studies were performed using NGS and microarray technology in thoroughbred horses [35, 40, 41]. These studies concentrated only on gene expression and genetic markers of athletic ability during and after exercise. Methylation analyses in animals exhibiting racing traits have not yet been reported. Many previous studies suggested that exercise induces methylation changes [42, 43], and athletic ability is closely associated with methylation [44, 45]. The regulation of methylation profiling related to exercise genes is important for exercising horses. Therefore, identifying methylation profiles related to exercise ability will be invaluable in studying athletic traits in racing horses. Nonetheless, there are no studies about the influence of methylation on the racing ability of TH let alone JH while the traits governing the economics of horse racing, such as the racing ability, speed, disease resistance, and recovery ability, are of important resource in the horse industry.

Here, we report the data and analyses of genome-wide DNA methylation patterns in the skeletal muscle, heart, lung, and cerebrum of TH and JH, and tissue-specific DNA methylation differences between the two horse breeds produced by methyl-DNA immunoprecipitation sequencing (MeDIP-seq).

## Results

### Global methylation analysis of thoroughbred and Jeju horses

We profiled the global DNA methylation status of physicality-associated organs (skeletal muscle, heart, lung, and cerebrum) of TH and JH using MeDIP sequencing. About 21 - 24 million raw reads from each samples were sequenced resulting in on average 820 K/mm2 of cluster density, producing about 1.05 - 1.2 Gbp. After low-quality data filtration, about 81.8% - 87.5% reads, assessed as clean data, were analyzed and mapped (Additional file 1: Table S1). On average, 17.5 and 16.0 million unique mapped reads were obtained from the four tissues of TH and JH, respectively, with a high-quality read lignment against the horse reference genome (Additional file 1: Table S1).

**Figure 1:**
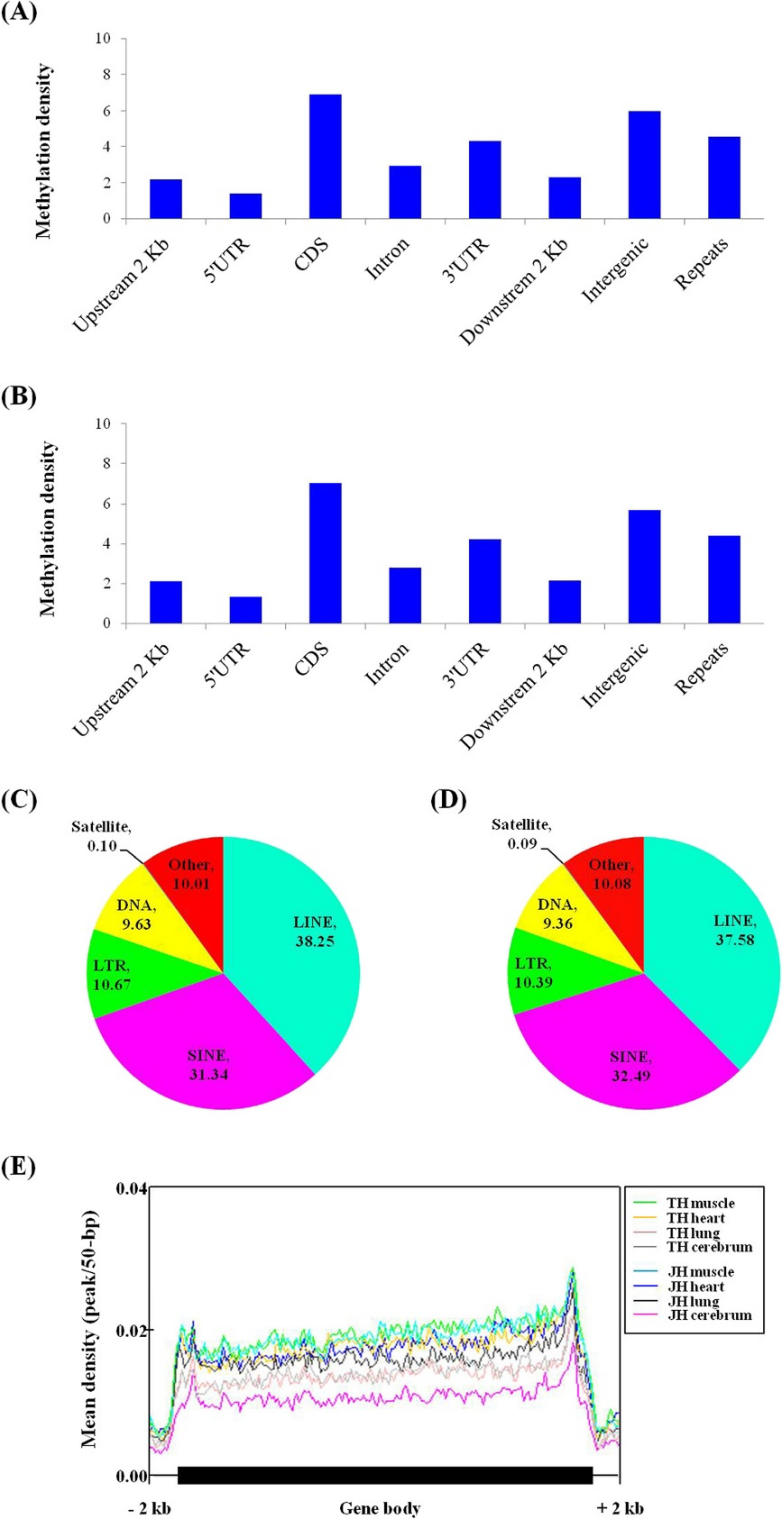
**The average methylation density in different genomic regions.** Methylation density within the gene regions, intergenic regions, and repeats were calculated by dividing the peak length in that region by the area of that region for thoroughbred **(A)** and Jeju **(B)** horse-derived DNA. Further repeats were classified in different classes and the average methylation level of each class was calculated in thoroughbred **(C)** and Jeju **(D)** horses. **(E)** Distribution of methylation density around gene body, including upstream to downstream 2 kb, was calculated for all RefSeq genes.

In the identification the global DNA methylation pattern, the number of methylated peaks in MeDIP-seq is important [46]. We obtained 61,000–112,000 methylated peaks in the TH and JH tissues (skeletal muscle, heart, lung and cerebrum), using the peak detection methodology which covers approximately 2.51-4.35% of the horse genome (2.7 Gbp) (Additional file 1: Table S1 and Table 1). These methylation peaks were observed with a moderate correlation of chromosomal length and gene number between methylation regions (Additional file 1: Figure S1). The degree of methylation was high in the intergenic regions containing repeats, followed by the intron and coding sequence (CDS) regions in both TH and JH (Table 1). However, the methylation density in the CDS region was higher than that in the intergenic region, whereas the methylation density in the other intragenic region such as 3’-UTR, intron, upstream 2 kb at transcription start site (TSS), and 5’-UTR was lower than that of the intergenic region (Figure 1A and 1B). Repeat elements showed a relatively high methylation density. In comparison with most of the repetitive elements, long interactive nuclear elements (LINE), short interactive nuclear elements (SINE), and long terminal repeat (LTR) elements exhibited a high level of methylation density in both TH and JH (Figure 1C, D). In this study, we demonstrated that the depletion or decrease of methylation density was found around TSS as well as promoter regions in both TH and JH, whereas the gradual increase of that was found in gene body (Figure 1E).

**Table 1:** **Peak distribution in different components of the thoroughbred horse and the Jeju horse**

Sample		Total peak number	Upstream 2 kb	5'UTR	CDS	Intron	3'UTR	Downstream 2 kb	Intergenic	Repeats
TH	Muscle	112,003	2,042	696	19,002	51,221	1,731	2,106	68,868	205,421
	Heart	96,574	1,788	704	18,021	43,829	1,634	1,852	58,892	161,360
	Lung	75,804	1,430	561	14,153	34,371	1,346	1,461	46,411	131,405
	Cerebrum	80,362	1,400	541	14,569	36,430	1,317	1,533	50,016	151,599
JH	Muscle	111,520	1,923	651	18,204	50,190	1,566	2,054	69,108	198,995
	Heart	97,477	1,801	719	18,327	44,541	1,693	1,828	59,563	171,184
	Lung	87,676	1,683	678	16,296	40,302	1,589	1,598	53,100	149,059
	Cerebrum	60,693	1,055	508	12,360	27,572	1,221	1,103	37,697	116,426

The methylation of CpG islands in the promoter regions is known to regulate gene expression and it was reported to be hypomethylated in the vertebrate genome [47]. The horse genome contained a total of 109,505 CpG islands. Of these CpG islands, about 12.3% (n = 13,467) were methylated in the skeletal muscle of TH, 7.65% (n = 8,377) in the heart of TH, 12.84% (n = 14,056) in the lung of TH, and 10.12% (n = 11,082) in the cerebrum of TH (Table 2). In addition, about 11.27% (n = 12,345) were methylated in the skeletal muscle of JH, 10.26% (n = 11,232) in the heart of JH, 12.73% (n = 13,939) in the lung of JH, and 7.82% (n = 8,560) in the cerebrum of JH. Therefore, we observed the most abundant CpG island methylation in the lung tissue in both TH and JH. Most of the methylated CpG islands were located in the intergenic regions in both the TH and JH. In the case of the gene body region, methylated CpG islands were present largely in the intron regions, followed by the CDS regions.

**Table 2:** **Summary of methylated CGIs in the different tissues of the thoroughbred and Jeju horses**

Sample		Upstream 2 kb	5'UTR	CDS	Intron	3'UTR	Downstream 2 kb	Other	Total methylated CGIs	Total CGIs	Methylated (%)
TH	Muscle	25	5	97	150	14	24	13,258	13,467	109,505	12.30
	Heart	22	7	62	83	10	16	8,245	8,377	109,505	7.65
	Lung	27	7	110	152	15	20	13,845	14,056	109,505	12.84
	Cerebrum	24	8	80	110	11	24	10,922	11,082	109,505	10.12
JH	Muscle	15	4	76	121	11	15	12,182	12,345	109,505	11.27
	Heart	23	4	88	114	14	20	11,072	11,232	109,505	10.26
	Lung	33	6	98	150	14	22	13,740	13,939	109,505	12.73
	Cerebrum	22	7	80	93	10	20	8,419	8,560	109,505	7.82

### Differential DNA methylation in thoroughbred and Jeju horses

**Figure 2:**
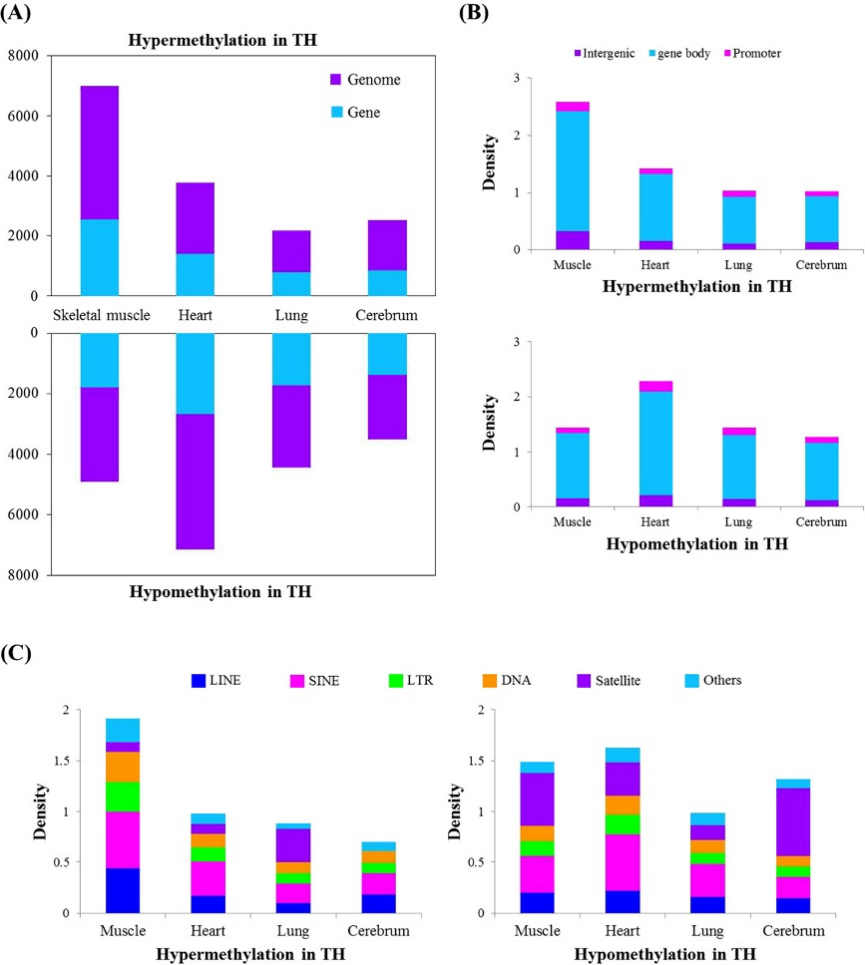
**Genomic distribution of differentially methylated regions (DMRs) in the thoroughbred horse compared to the Jeju horse. (A)** The number of hyper- and hypomethylated DMRs in 4 different tissues of thoroughbred horses. **(B)** Distribution of hyper- and hypomethylation density in different genomic regions such as intergenic, gene body, and promoter regions. **(C)** Hyper- and hypomethylation density in repeat regions, classified according to the family.

**Figure 3:**
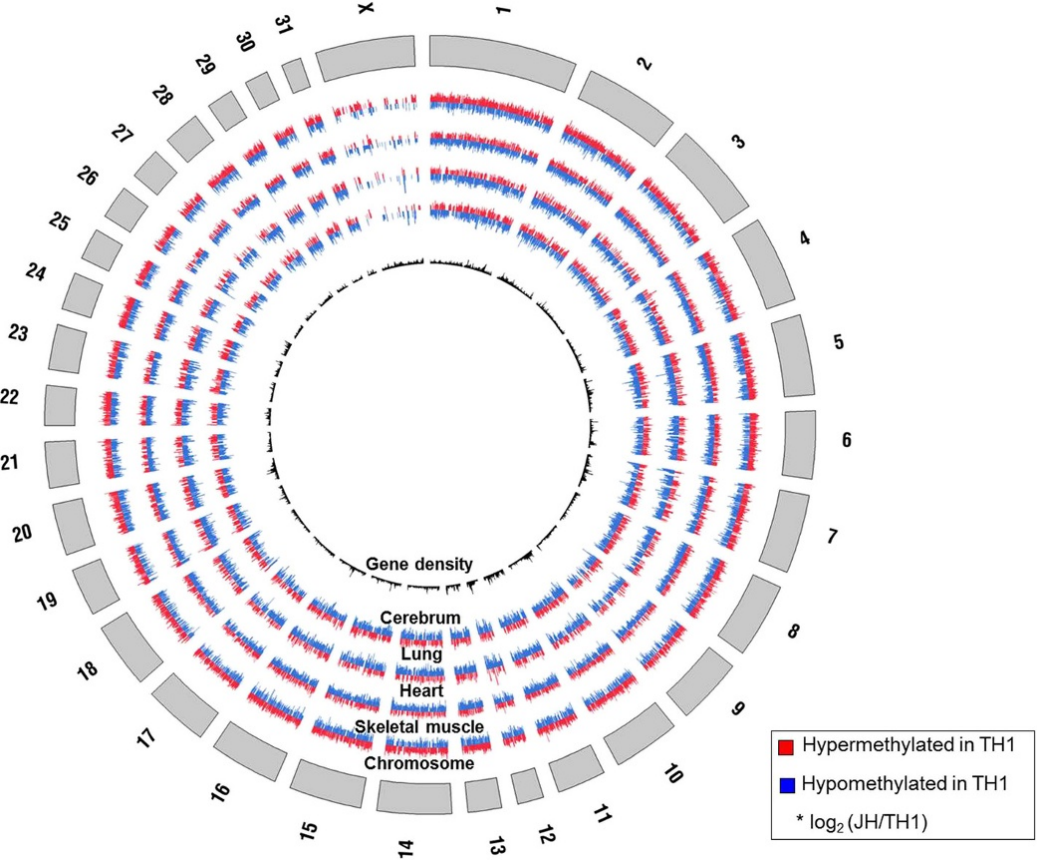
**Comprehensive maps of the entire DNA methylome of thoroughbred and Jeju horses.** Circular representation of the hyper- and hypomethylation levels for four different tissues of thoroughbred horse.

We observed a total of 35,467 differentially methylated regions (DMRs) in the four different TH and JH tissues, indicating differences in their methylation profiles (Additional file 1: Table S2). The TH’s skeletal muscle was hypermethylated compared to that of JH, whereas the heart, lung, and cerebrum of TH showed a hypomethylated pattern compared to those of JH (Figure 2A). We also analyzed methylation events in the intergenic, gene body, and promoter regions in the four tissues of TH and JH. As shown in Figure 2B, the gene body region in the skeletal muscle of TH showed a relatively high level of methylation, whereas the gene body in the heart of TH showed a high hypomethylation pattern, compared to other tissues. We also examined DMRs within the repeat region, and found that SINE and LINE elements showed a high level of methylation in skeletal muscle compared to that of JH. The satellite regions indicated a high hypermethylation density in lung tissue compared to that of JH (Figure 2C). Here, based on our DMR data, we provide the DMRs associated with comprehensive maps of the DNA methylome of TH and JH (Figure 3).

### MeDIP-seq data validation

**Figure 4:**
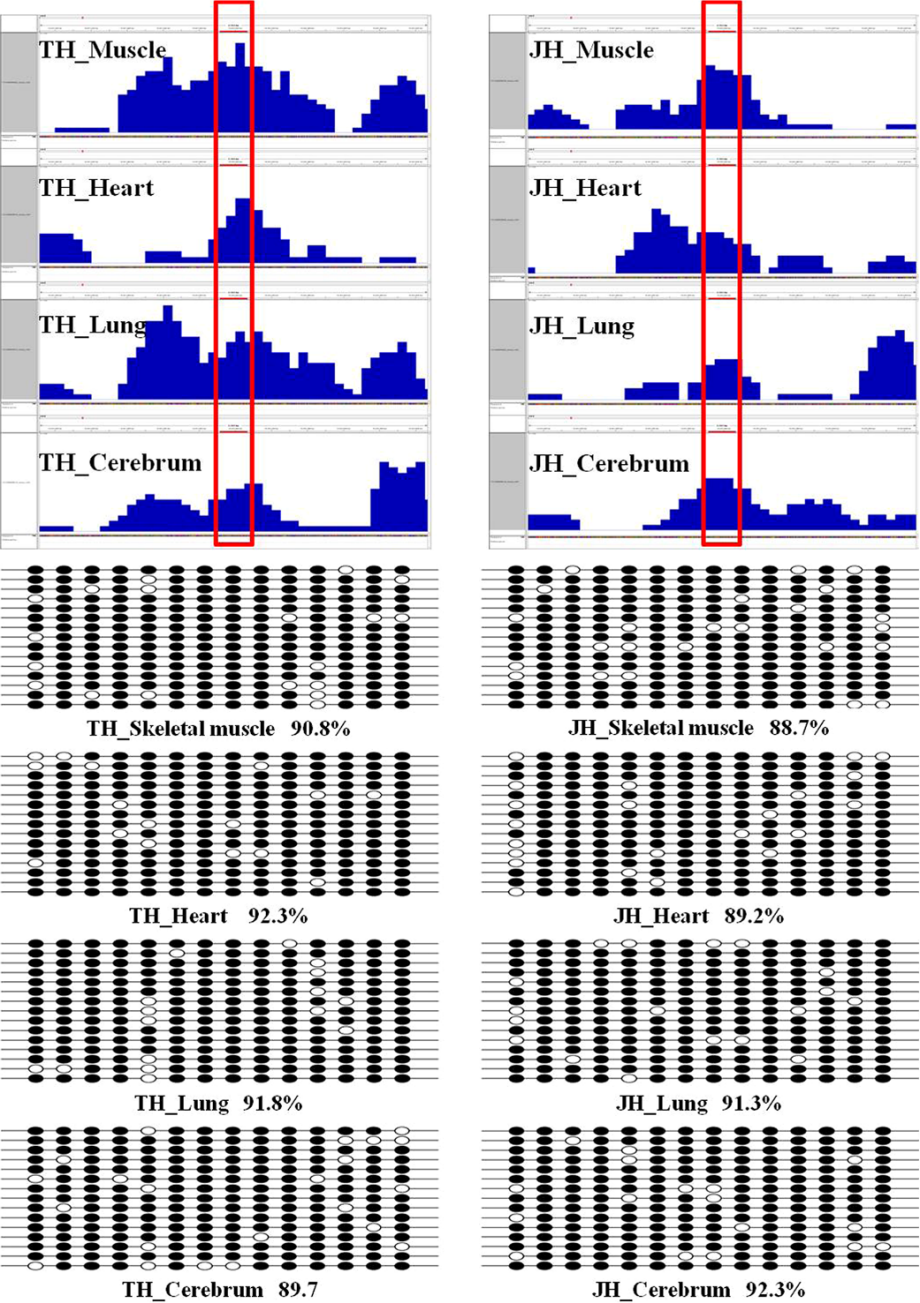
**The validation of MeDIP-seq data by bisulfite sequencing (BSP).** A high methylated region obtained from MeDIP-seq data was selected randomly and its methylation pattern was profiled by BSP. The box indicated amplification regions. CpG dinucleotides are represented by circles on vertical bars. Each line represents an independent clone, and methylated CpGs are marked by filled circles, unmethylated CpGs by open circles.

To validate the results obtained with MeDIP-seq data, three regions were selected in the horse genome for analysis by bisulfite sequencing. We randomly chose one region with a relatively high level of methylation, one region with a moderate level of methylation and one region of differential methylation region between TH and JH. The bisulfite sequencing results showed a high degree of consistency with the MeDIP-seq data (Figure 4, Additional file 1: Figure S2, and Additional file 1: Figure S3). These results indicated that our genome-wide methylation results obtained by MeDIP-seq are reliable.

### Analysis of functional categories of DMR-containing genes

**Figure 5:**
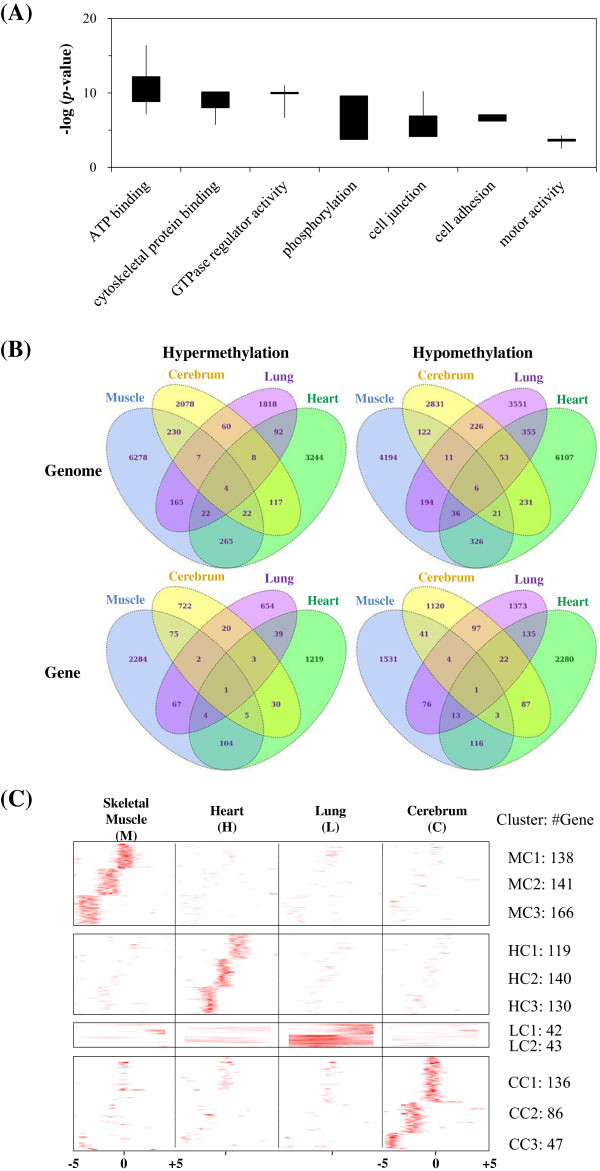
**Functional classification and comparison of differentially methylated regions (DMRs). (A)** GO analysis of biological function. **(B)** The Venn diagram for comparison of DMRs that are unique or shared in four tissues derived from thoroughbred and Jeju horses. **(C)** *k*-mean clustering (k = 5) analysis of differential methylated genes.

To explore the biological functions associated with DMR-containing genes in the thoroughbred horse, we analyzed the gene ontology (GO) categories of these genes using DAVID (http://david.abcc.ncifcrf.gov/) [48]. All genes analyzed with GO annotations were used as the reference list. We selected some categories associated with exercise ability in the horse [49]. Several categories were related to exercise ability; however, we chose the category sets associated with overexpression and tissue capacity functions (Figure 5A). Comparison of gene methylation showed that there were 12,128 DMRs among TH and JH. DMRs and genes that are unique or shared among the four tissue types examined are shown in Figure 5B. Genes having high numbers of DMRs are dominant in the muscle (4327) and heart (4062). These two tissues have more DMR-containing genes than the cerebrum and lung; in particular, TH’s muscle tissue has the highest number of hypermethylated DMR-containing genes among the four tissues analyzed. The frequency of hypomethylation in the cerebrum, lung, and heart tissues was higher in TH than the JH.

Tissue-specific DMRs were identified by *k*-mean clustering in the methylation regions in the four tissues. Several genes containing DMRs were clustered, and were divided into 11 clusters (Figure 5C). The k-mean clustering of 1188 genes revealed differential methylation in each tissue (P = 0.0005 ~ 0.00051 for skeletal muscle-related clusters (MC1-MC3), P = 0.0005 for heart-related clusters (HC1-HC3), P = 0.00128 for lung-related clusters (LC1 and LC2), and P = 0.017 ~ 0.015 for cerebrum-related clusters (CC1-CC3)). Clusters of tissue-specific DMRs were located upstream of the TSS, which is 5 kb upstream of genes in the skeletal muscle and cerebrum. However, in heart and lung tissues, each cluster of DMRs was evenly spread over the region upstream and downstream of the TSS site. In heart tissue, tissue-specific DMR clusters were detected in several genes, while in the lung tissue, tissue-specific DMR clusters were detected in 85 genes.

## Discussion

We report the analyses and data generated by methyl-DNA immunoprecipitation sequencing to provide the genome-wide DNA methylation patterns in skeletal muscle, heart, lung, and cerebrum tissues of TH and JH. In the horse genome, gene body regions showed a higher methylation density than the intergenic regions. Also the repetitive elements had a high methylation density while CpG islands showed a low methylation density. These patterns revealed in this study were similar to those previously reported in other species, from plants to mammalians [13, 17, 50].

The promoter and 5'-UTR regions play an important role in the regulation of gene expression and they have been reported to be hypomethylated [51]. In the case of the gene body region, except for the 5'-UTR, DNA methylation contributed to chromatin structure alteration and regulation of the transcription elongation efficiency [52]. We report an increased level of methylation in the CDS, intron, and 3'-UTR regions in TH and JH, these results are similar to those from previously reported animal studies [22, 28]. Repeat elements occupied about 30–50% of the mammalian genome; among these, LINE elements were predominantly interspersed. In the horse genome, LINE elements were also the most predominantly interspersed repeat elements [53]. Repeat elements are usually associated with genomic instability through structural changes such as transposition, translocation, and recombination [54, 55]. To maintain genomic stability, DNA methylation functions as a silencing mechanism for repeat elements [56]. Thus, a major proportion of genomic methylation has been reported to occur in repeat elements, which is supported by our data. We found that DNA methylation was predominantly seen in LINE elements, consistent with findings from previous animal studies [47]. Additionally, SINE and LTR elements were hypermethylated in the horse genome, similar to the results in other animal studies [47, 57]. Methylation of these elements is known to be a crucial factor in the maintenance of genomic stability through the suppression of transcription, transposition, and recombination [17]. Thus, hypermethylation of repeat elements in the horse genome might play an essential role, as a defense mechanism to maintain genomic stability in the presence of active repeat elements. CpG islands have been universally reported to be regions of gene regulation via methylcytosine, possibly through the mechanism of transcriptional repression. These regions in the mammalian genome are known to be generally demethylated, in spite of having a high GC content [4]. Intragenic and intergenic methylated CpG islands affect functional gene expression through the regulation of promoter activity, and intergenic methylated CpG islands play a crucial role in the regulation of alternative promoters and splicing [48]. In this study, we found that about 10.73% and 10.52% of the CpG islands were methylated in TH and JH genomes, respectively, which is similar to that observed in the human genome (about 6–8%) [8]. Further analysis of the density of methylated CpG islands in intragenic regions showed a higher methylation level in exons (11.06 ± 1.78) than in introns (1.28 ± 0.28) in the horse genome. These results were consistent with the findings in humans and rats [8, 17]. Taken together, we provide a comprehensive data and information of the whole methylome in horse, They can enable researchers to perform in depth analyses of the roles played by DNA methylation in horses and probably in other mammals.

DNA methylation is one of the main epigenetic modification mechanisms; thus, the study of DMRs within tissues or individual organisms is important. In several studies, various levels of DNA methylation could regulate tissue-specific transcription and may be important during development and differentiation [58]. Thus, the analysis of DMRs among tissues is essential in understanding tissue specific gene expression. In particular, methylation analysis between breeds in a well-known subspecies can provides invaluable information on the evolutionary divergence and evidence for useful traits. We successfully identified differentially methylated regions within four tissues in two horse breeds. Similar results have been reported in pig tissues from various breeds [59, 60] that can be compared. Differential methylation patterns were observed in seven tissues (muscle, heart, liver, spleen, lung, kidney, and stomach) from Laiwu, a specific pig breed [60]. In addition, the level of methylation in the liver tissue genome of other breeds of pigs (such as Berkshire, Duroc, and Landrace) also differed [59]. Distribution patterns of DNA methylation are generally conserved among these three pig breeds, but some DMRs were detected in the coding genes and repetitive element regions in liver tissue. In this study, we also observed that distribution of DNA methylation in the two breeds showed generally conserved pattern but, some DMRs were detected a high density in the gene body, including the coding regions and introns. Gene body methylation, especially intronic DNA methylation, may be associated with alternative splicing [61]. Thus, these results suggest that methylation has important effects on gene transcription in individual breeds. Furthermore, in the repeat region, the density of DMRs was dominant. Thus, the high density of DMRs in repeat regions could also induce differences in transcript variation and expression. In summary, differences in DNA methylation patterns and the density of DMRs in the four tissues of individual breeds may play a crucial role in the process of development and the corresponding gene expression.

Gene containing DMRs in the tissues of TH showed high representation in the categories of ATP binding and cytoskeletal protein binding. ATP binding functions play a role during exercise, as they affect ATPase activity. ATPase activity-induced ATP lysis subsequently caused intermediate molecular interactions using the energy of ATP lysis [60]. In TH, these functions may play important roles and the dominant expression of these gene categories is required. In particular, during exercise, ATP binding could induce muscle contraction [62]. After ATP binds the myosin head, muscle contractions are initiated due to the detachment of myosin from actin filaments [63]. DMRs in the tissues of TH are also overrepresented in cytoskeletal protein binding. Generally, the cytoskeleton plays important roles in both intracellular transport and cellular division [63]. In eukaryotic cells, the cytoskeleton can be classified into three types: microfilaments, intermediate filaments, and microtubules [64]. Muscle activity-related units such as actin, keratin, and tubulin are included in the cytoskeleton. Thus, genes having DMRs could influence their binding and activities, thus differentially affecting the exercise ability in TH. These functional categories of genes with DMRs suggest that DNA methylation has an important effect on the regulation of genes categorized as being involved in ATP and cytoskeletal binding. Thus, these differences in methylation status in the tissues of TH and JH may indicate differences in their gene expression levels and exercise ability characteristics.

## Conclusions

We have generated, for the first time, DNA methylomes for TH and JH. We provide the DNA methylation landscape and differentially methylated genomic regions in these horse species, indicating that DMRs represent comprehensive maps of the DNA methylome in TH and JH. These DNA methylome maps could be useful for further studies of epigenetic gene regulation in various horse breeds. The epigenetic system existing in the horse genome lays the foundation for studying the involvement of epigenetic modifications in horse domestication and improvement and provides a more systemic analysis of DNA methylation.

## Methods

### Ethics statement

The animal protocol used in this study has been reviewed by the Pusan National University-Institutional Animal Care and Use Committee (PNU-IACUC) on their ethical procedures and scientific care, and it has been approved (Approval Number PNU-2013-0411).

### Genomic DNA extraction

The healthy thoroughbred (retired racing horse, Korea Racing Authority registered number: 016222; 5 years old; a castrated horse) and Jeju horses (tested Jeju native horse breed registered number: P06071M1; 6 years old; male) were sacrificed in compliance with the international guidelines for experimental animals, and the tissues were separated and stored at -80°C. The use of these samples was approved by the National Institute of Subtropical Agriculture in Jeju Island, South Korea. Genomic DNA was isolated from 4 tissue samples from each healthy horse (skeletal muscle, heart, lung, and cerebrum) using the DNeasy Blood & Tissue Kit (Qiagen, Hilden, Germany) according to the manufacturer’s manual for MeDIP-Seq and bisulfite-treatment experiments. DNA concentration and quality were estimated by UV spectrophotometry on a NanoDrop ND-1000 (NanoDrop, Wilmington, DE, USA). For quality control, we selected only those DNA samples in which the A260/A280 ratio range was 1.6 to 2.2, the A260/A230 ratio was >1.6, and the main band was identified by agarose gel electrophoresis.

### Methyl-DNA immunoprecipitation sequencing

Eight genomic DNA samples with 1 μgof genomic DNA starting material (DNA concentration of 0.1 μg/μl) were sonicated to produce DNA fragments ranging from 100 to 500 bp. After DNA end-repair and the generation of 3'-dA overhangs using the Paired-End DNA Sample Prep Kit (Illumina, San Diego, CA, USA), the DNA samples were ligated to Illumina sequencing adaptors. The fragments were denatured and then immunoprecipitated using magnetic methylated DNA immunoprecipitation kit including a 1:10 diluted antibody mix (0.3 ul antibody, 0.6 ul buffer A, and 2.10 ul distilled water) following the manufacturer’s recommendation (Diagenode, Delville, NJ, USA). The immunoprecipitated DNA was quantified by quantitative real-time PCR (qPCR). DNA fragments between 200 and 300 bp were excised from the gel and purified using a gel extraction kit (Qiagen). The products were quantified with a Quant-iTTM dsDNA High Sensitivity Assay Kit (Invitrogen, Carlsbad, CA, USA) on an Agilent 2100 Analyzer (Agilent Technologies, Santa Clara, CA, USA). After qPCR analysis, DNA libraries were subjected to paired-end sequencing with a 50-bp read length using the Illumina HiSeq 2000 platform (Illumina). After the completion of a sequencing run, raw image files were processed by Illumina Real-Time Analysis (RTA) for image analysis and base calling. Sequencing reads have been submitted to the NCBI Short Read Archive (SRA) under an SRA accession no.SRP041333.

### Bioinformatics analysis

Raw sequence data were first processed to filter out adapters and low-quality reads with the follow criteria; (1) N’s per read ≥ 10%, (2) average of quality score (QS) per read < 20, (3) number of nucleotides with < QS 20 per read ≥ 5%, and (4) having called the same bases in paired-end reads. The filtered data were then aligned to the horse reference genome (EquCab2) using the SOAPaligner (version 2.21) with mismatches of no more than 2 bp [65]. Uniquely mapped reads were retained for further analyses. To identify genomic regions that are enriched in a pool of specifically immunoprecipitated DNA fragments, genome-wide peak scanning was carried out by MACS (version 1.4.2) with a cutoff of *P*-value of 1 × 10^-4^ to exclude false positive peaks or noises [66]. In addition, an option of ‘--mfold’ to select the regions with MFOLD range of high-confidence enrichment ration against background to build model was used with lower limit 10 and upper limit 30. The distribution of peaks in different regions of the horse genome in each sample, including the promoter, 5'-untranslated region (UTR), 3'-UTR, exons, introns, intergenic regions, CpG islands (CGIs), and repeats, was analyzed. Methylated peaks corresponding to different genomic regions were selected by mapping at least 50% of the peak on a particular genomic region. In particular, CGI can be defined by 3 criteria: length greater than 200 bp, ≥50% GC content, ≥0.6 of CpG observed/expected ≥0.6. The methylation densities in the different regions of the genome were also compared.

To identify candidate differentially methylated regions (DMRs) in any 2 samples, their peaks were merged, and the number of reads within those peaks were assessed with chi-square and FDR statistics (*P* < 0.05). DMRs with a greater than 2-fold difference in read numbers were finally selected and classified as hyper- or hypo-methylated regions. All DMR-containing genes were used for subsequent gene ontology (GO) enrichment analyses using the DAVID Functional Annotation Tool with *P* < 0.05 [49]. Moreover, co-existing DMRs within genes among different tissues were plotted and centered at a transcription start site (TSS) using seqMINER with the k-mean clustering method [67].

### Bisulfite sequencing (BSP)

Three pairs of primers (Additional file 1: Table S3) were designed with MethPrimer tool (http://www.urogene.org/cgi-bin/methprimer/methprimer.cgi), including one pair for the validation of relatively high methylated region, one pair for relatively moderate methylated region, and one pair for differentially methylated regions between TH and JH. Bisulfite modification of 1 μg of genomic DNA was performed using the Imprint® DNA Modification kit by standard methods (SIGMA). The bisulfite-treated DNA was amplified by PCR with BSP specific primer pair. After a hot start, PCRs were carried out for 40 cycles of 94°C for 40 sec, 50-55°C for 40 sec, and 72°C for 40 sec. PCR products were separated on a 1.5% agarose gel, purified with the LaboPass gel extraction kit (COSMO GENETCH) and cloned into the pGEM-T-easy vector (Promega). The cloned DNA was isolated using the Plasmid DNA mini-prep kit (GeneAll). Positive clones were randomly collected for sequencing at COSMO GENETCH company (Seoul, Korea).

## Availability of supporting data

All sequencing reads from this study have been submitted to the NCBI Sequence Read Archive; SRA (http://www.ncbi.nlm.nih.gov/sra/) under accession no. SRP041333.

## Electronic supplementary material

Additional file 1: Figure S1: Pearson’s correlation between methylated peaks, chromosome length, and gene number. The peaks were plotted against chromosome length **(A)** and gene number **(B)**. **Figure S2.** Validation of MeDIP-seq data by bisulfite sequencing with relatively moderate methylated region. Box indicated amplification regions. CpG dinucleotides are represented by circles on vertical bars. Each line represented an independent clone, and methylated CpGs are marked by filled circles, unmethylated CpGs by open circles. **Figure S3.** Validation of MeDIP-seq data by bisulfite sequencing with differentially methylated regions in skeletal muscle. Box indicated amplification regions. CpG dinucleotides are represented by circles on vertical bars. Each line represented an independent clone, and methylated CpGs are marked by filled circles, unmethylated CpGs by open circles. **Table S1.** The general information of MeDIP-seq data in different each tissues from the Thoroughbred and Jeju horse. **Table S2.** Comparison of the number of differentially methylated region in throughbred and Jeju horse. **Table S3.** The information of primers for BSP. (DOCX 590 KB)
