# Supplementary material for: Genome-wide analysis of DNA methylation patterns in horse
Source: BMC Genomics. 2014 Jul 15;15(1):598. doi: 10.1186/1471-2164-15-598 (PMC4117963; doi:10.1186/1471-2164-15-598)
Supplement: Supplementary file 1 — Additional file 1: Figure S1: Pearson’s correlation between methylated peaks, chromosome length, and gene number. The peaks were plotted against chromosome length (A) and gene number (B). Figure S2. Validation of MeDIP-seq data by bisulfite sequencing with relatively moderate methylated region. Box indicated amplification regions. CpG dinucleotides are represented by circles on vertical bars. Each line represented an independent clone, and methylated CpGs are marked by filled circles, unmethylated CpGs by open circles. Figure S3. Validation of MeDIP-seq data by bisulfite sequencing with differentially methylated regions in skeletal muscle. Box indicated amplification regions. CpG dinucleotides are represented by circles on vertical bars. Each line represented an independent clone, and methylated CpGs are marked by filled circles, unmethylated CpGs by open circles. Table S1. The general information of MeDIP-seq data in different each tissues from the Thoroughbred and Jeju horse. Table S2. Comparison of the number of differentially methylated region in throughbred and Jeju horse. Table S3. The information of primers for BSP. (DOCX 590 KB) [file 12864_2013_6300_MOESM1_ESM.docx]

**Supporting Information**

**
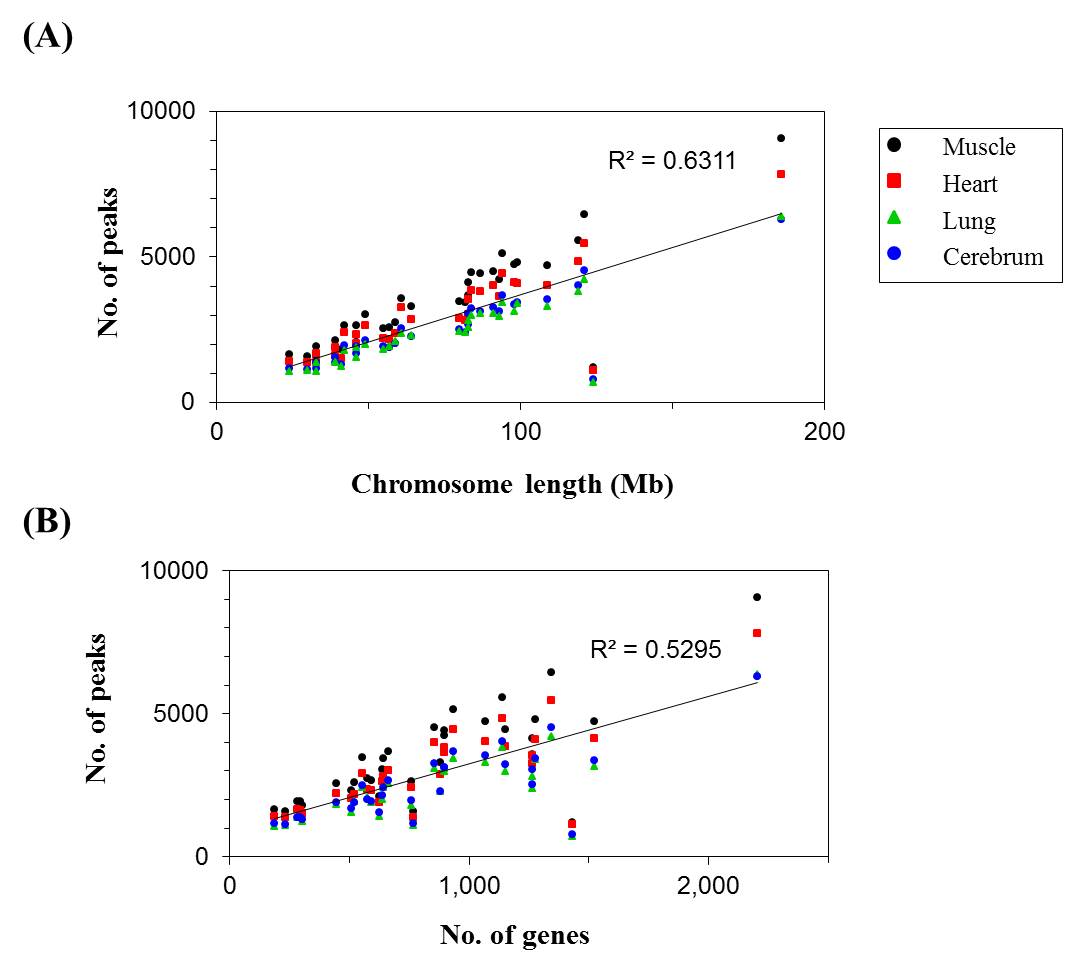
**

**Figure S1.** **Pearson’s correlation between methylated peaks, chromosome length, and gene number.** The peaks were plotted against chromosome length (A) and gene number (B).


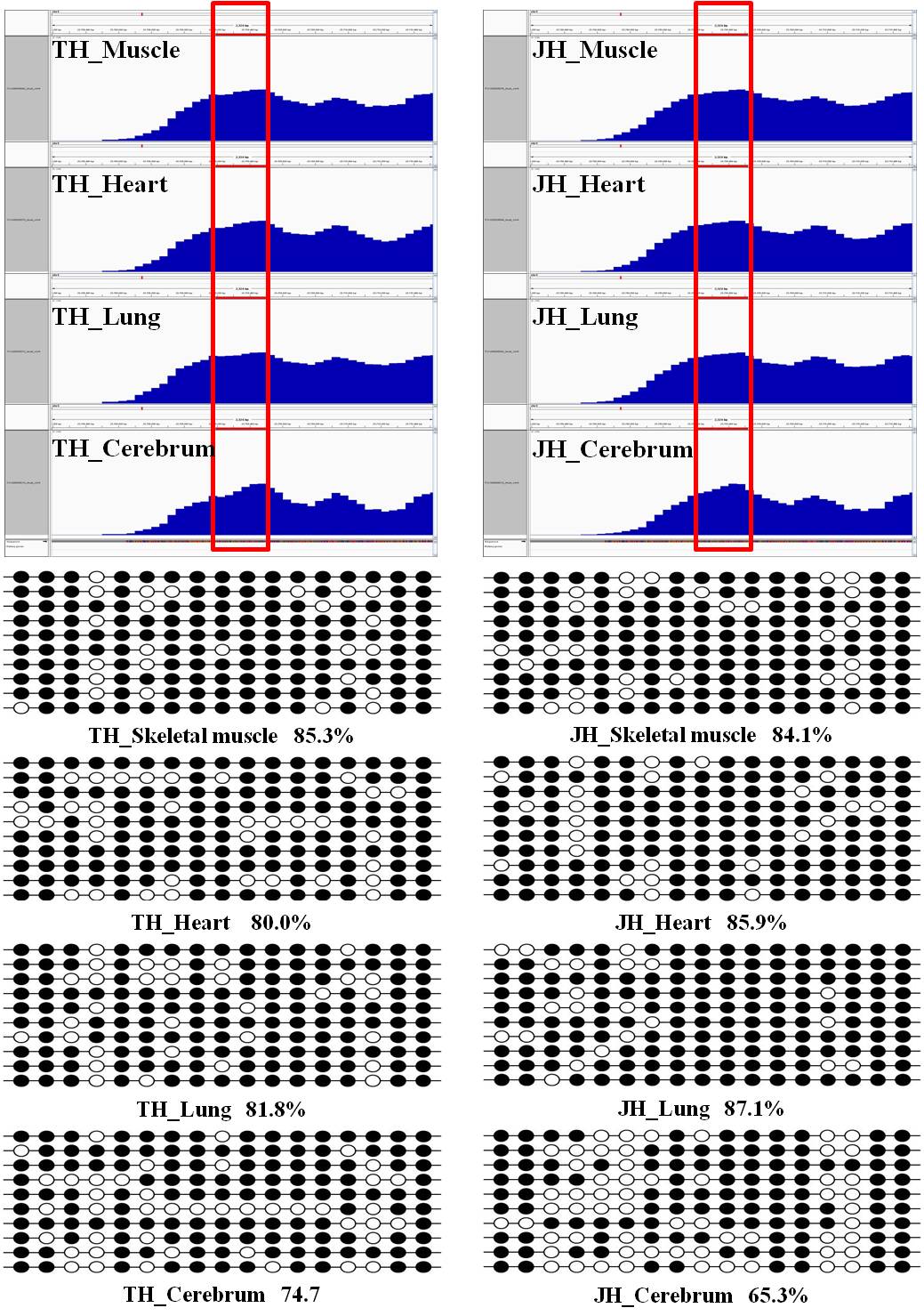


**Figure S2. Validation of MeDIP-seq data by bisulfite sequencing with relatively moderate methylated region.** Box indicated amplification regions. CpG dinucleotides are represented by circles on vertical bars. Each line represented an independent clone, and methylated CpGs are marked by filled circles, unmethylated CpGs by open circles.


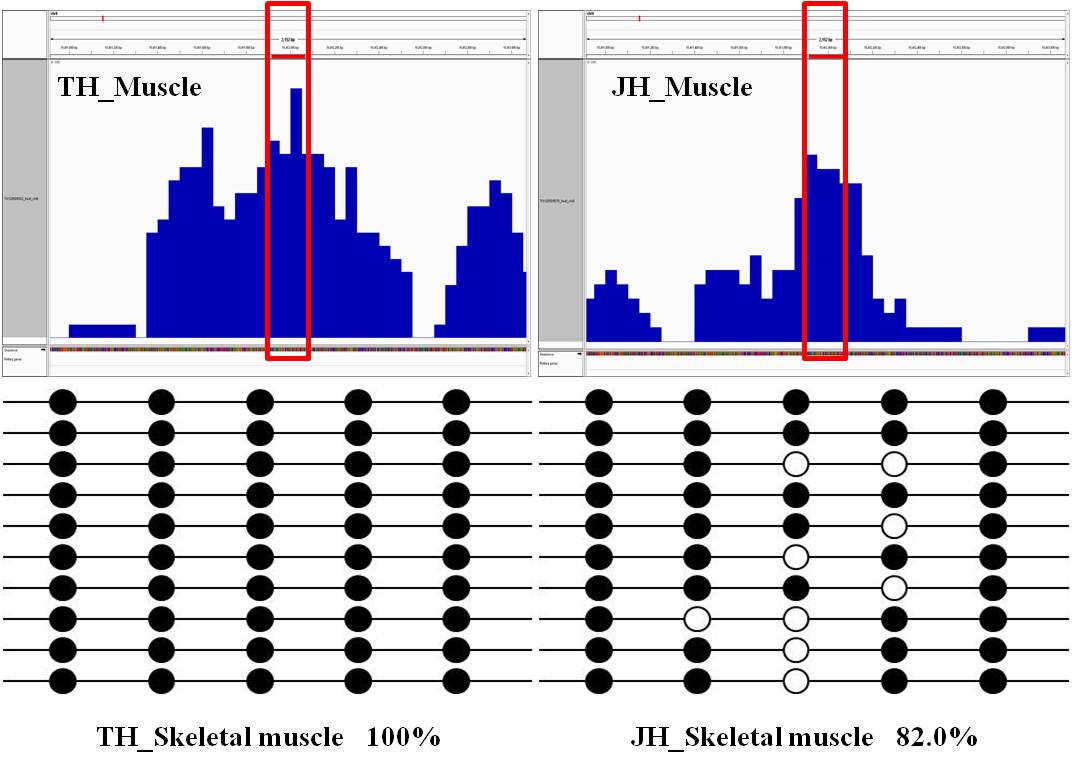


**Figure S3.** **Validation of MeDIP-seq data by bisulfite sequencing with differentially methylated region in skeletal muscle.** Box indicated amplification regions. CpG dinucleotides are represented by circles on vertical bars. Each line represented an independent clone, and methylated CpGs are marked by filled circles, unmethylated CpGs by open circles.

**
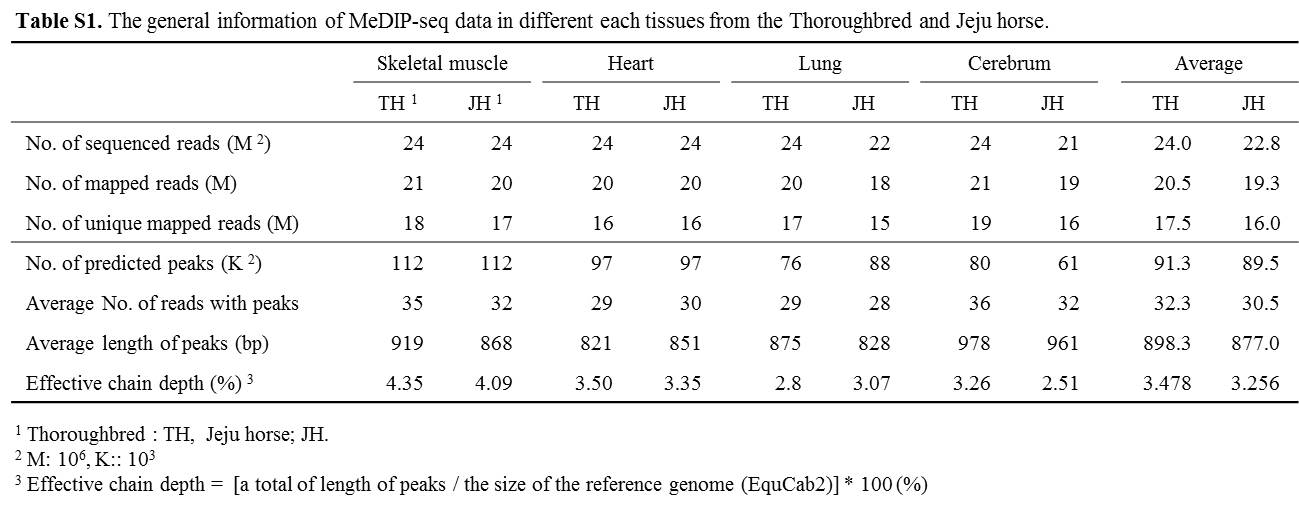
**

| **Table S2.** Comparison of the number of differentially methylated regions in thoroughbred and Jeju horses. | | | | |
| --- | --- | --- | --- | --- |
|  | | **Gene** | **Genome** | **Total** |
| **Hypermethylation** | **Skeletal muscle** | 2542 | 4451 | 6993 |
|  | **Heart** | 1405 | 2370 | 3775 |
|  | **Lung** | 790 | 1386 | 2176 |
|  | **Cerebrum** | 858 | 1669 | 2527 |
| **Hypomethylation** | **Skeletal muscle** | 1787 | 3126 | 4913 |
|  | **Heart** | 2659 | 4487 | 7146 |
|  | **Lung** | 1721 | 2712 | 4433 |
|  | **Cerebrum** | 1376 | 2128 | 3504 |

| **Table S3. The information of primers for BSP.** | | | | | |
| --- | --- | --- | --- | --- | --- |
| **Primers** | **Primer sequence** | **Length (bp)** | **GpG**  **site** | **AT^1^**  **(℃)** | **Location** |
| P1 | F: 5'-AAG GAT ATT TAG ATA GTA TTT TAG GA-3' | 219 | 13 | 51 | chr11:37,716,808-37,717,026 |
|  | R: 5'-ATT CTA ATC ACC AAT TTC CAC C-3' |  |  |  |  |
| P2 | F: 5'-TTT GGT TAA GAT TTG GTT GGT A-3' | 327 | 17 | 51 | chr4:25,709,192-25,709,518 |
|  | R: 5'-AAC CCA AAC CTA AAA CAC ATA TCT AC-3' |  |  |  |  |
| P3 | F: 5'-TGG TTT AAA TAT ATT TTA AGA GGA A-3' | 148 | 5 | 55 | chr8:10,411,920-10,412,067 |
|  | R: 5'-AAA CCC AAA TAA AAA ACC AC-3' |  |  |  |  |
| ^1^ Anealing temperature. | | | | | |
